# Supplementary material for: Molecular Characterization of a DNA Polymerase from Thermus thermophilus MAT72 Phage vB_Tt72: A Novel Type-A Family Enzyme with Strong Proofreading Activity
Source: Int J Mol Sci. 2022 Jul 19;23(14):7945. doi: 10.3390/ijms23147945 (PMC9324360; doi:10.3390/ijms23147945)
Supplement: Supplementary file 1 [file ijms-23-07945-s001.zip › ijms-1792693-supplementary.pdf]

## Supplementary Materials

**Table S1.** Sequences of primers and oligonucleotides used in this study.

| Primer name | Oligonucleotide sequence (5′→3′) | Tm |
|---|---|---|
| **Cloning of *polTt72* gene to expression vectors** | | |
| polTt72-NdeI-F | CGAATTCCATATGGACAAAGACCTTTTGGGATAC | 62.0 °C |
| polTt72-BamHI-R | CGCGGATCCTTAAAGAACATTATTAAAACATTCTTCAC | 61.2 °C |
| polTt72-SalI-R | CGCGTCGACAAGAACATTATTAAAACATTCTTCAC | 61.0 °C |
| **Cloning of *polTt72* gene to pHSG576** | | |
| polTt72-PstI-F | CGATTCCTGCAGCTATGGACAAAGACCTTTTGGGATAC | 66.6 °C |
| polTt72-BamHI-R | CGCGGATCCTTAAAGAACATTATTAAAACATTCTTCAC | 61.2 °C |
| **Cloning of *polTth* gene to pHSG576** | | |
| polTth-SalI-F | CGATTCGTCGACATATGGAGGCGATGCTTCCGCTCTTTGAACCCA | 71.8 °C |
| polTth-EcoRI-R | GCGGAATTCCTAACCCTTGGCGGAAAGCCAGTCCTCCCCCATCC | 74.8 °C |
| **Cloning of *polTaq* gene to pHSG576** | | |
| polTaq-SalI-F | CGATTCGTCGACATATGAGGGGGATGCTGCCCCTCTTTGAG | 71.5 °C |
| polTaq-EcoRI-R | GCGGAATTCTCACTCCTTGGCGGAGAGCCAGTCCTC | 71.3 °C |
| **Terminal transferase activity assay** | | |
| polTt72-Cy3 | Cy3-TGGCTGCTTCTAAGCCAACATCCT | 57.4 °C |
| polTt72-blunt | AGGATGTTGGCTTAGAAGCAGCCA | 57.4 °C |
| **Microscale thermophoresis** | | |
| polTt72-Cy5 | Cy5-TGGCTGCTTCTAAGCCAACATCCT | 57.4 °C |
| polTt72-long | CTAGTGAGGATGTTGGCTTAGAAGCAGCCA | 63.0 °C |

**Table S2.** Sequences of primers used in site-directed mutagenesis of *polTt72* gene.

| Primer name: | Oligonucleotide sequences (5′→3′): | Codon change: |
|---|---|---|
| D15A_F | ATACAAAAAGATTGTTGGGATAGCTATAGAAACATGGGACGGTAAAC | GAT → GCT |
| D15A_R | GTTTACCGTCCCATGTTTCTATAGCTATCCCAACAATCTTTTTGTAT | GAT → GCT |
| E17A_F | GATTGTTGGGATAGATATAGCAACATGGGACGGTAAACGAG | GAA → GCA |
| E17A_R | CTCGTTTACCGTCCCATGTTGCTATATCTATCCCAACAATC | GAA → GCA |
| L27A_F | GGGACGGTAAACGAGGAGGATCAGCTGATCCTTATTATG | CTT → GCT |
| L27A_R | CATAATAAGGATCAGCTGATCCTCCTCGTTTACCGTCCC | CTT → GCT |
| D78A_F | GTTGGGCACAACCTAAAGTTTGCCCTTAAGTTCTTTTATATGAACA | GAC → GCC |
| D78A_R | TGTTCATATAAAAGAACTTAAGGGCAAACTTTAGGTTGTGCCCAAC | GAC → GCC |
| Y180A_F | GTGAAGCACAAAAAGAGGCCGCAATCAACGATGTTAAG | TAC → GCC |
| Y180A_R | CTTAACATCGTTGATTGCGGCCTCTTTTTGTGCTTCAC | TAC → GCC |

| | | |
|---|---|---|
| D184A_F | CACAAAAAGAGTACGCAATCAACGCTGTTAAGTACTTAAAAGATTTAGC | GAT → GCT |
| D184A_R | GCTAAATCTTTTAAGTACTTAACAGCGTTGATTGCGTACTCTTTTTGTG | GAT → GCT |
| D384A_F | AGGCTATGTTCCTGTATTTGCTGCTTATTCTCAAGTAG | GAT → GCT |
| D384A_R | CTACTTGAGAATAAGCAGCAAATACAGGAACATAGCCT | GAT → GCT |
| E389A_F | GCTGATTATTCTCAAGTAGCATTAAGAATTCTTGCC | GAA → GCA |
| E389A_R | GGCAAGAATTCTTAATGCTACTTGAGAATAATCAGC | GAA → GCA |
| D615A_F | GGTATATGACATTGACAGTTCACGCTAGTATCTTTTTTGAG | GAT → GCT |
| D615A_R | CTCAAAAAAGATACTAGCGTGAACTGTCAATGTCATATACC | GAT → GCT |
| S616A_F | GGTATATGACATTGACAGTTCACGATGCTATCTTTTTTGAG | AGT → GCT |
| S616A_R | CTCAAAAAAGATAGCATCGTGAACTGTCAATGTCATATACC | AGT → GCT |

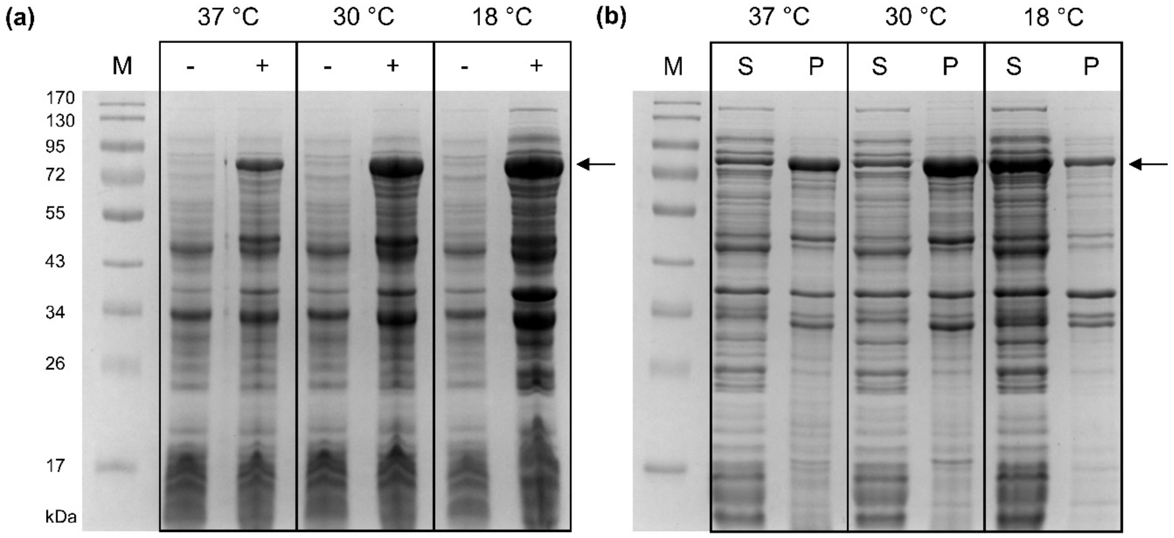


**Figure S1.** (**a**) Effect of temperature on overproduction of the Tt72 pol in *E. coli* Bl21(DE3)[pRARE][pET15b_polTt72]; (**b**) Solubility analysis of recombinant protein. Proteins were separated in 10% SDS-polyacrylamide gel electrophoresis and stained with Coomassie Brilliant Blue. An arrow indicates the position of the His-tagged recombinant Tt72 pol (82,650 Da); lane (-) before induction; (+) after induction with IPTG (1 mM); S, soluble fraction; P, insoluble fraction; M, protein molecular mass markers (PageRuler™ Prestained Protein Ladder, Thermo Scientific).

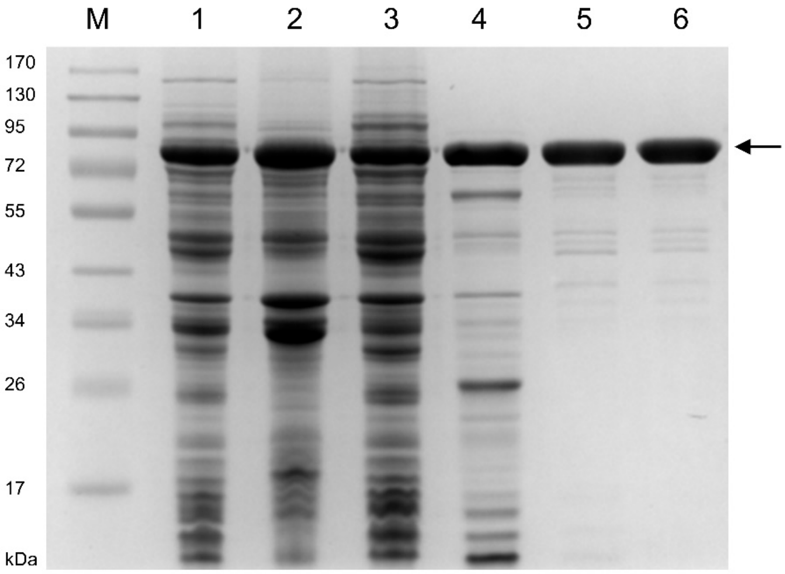


**Figure S2.** Successive steps in purification of the Tt72 pol. Proteins were separated in 10% SDS-polyacrylamide gel electrophoresis and stained with Coomassie Brilliant Blue. An arrow indicates the position of the His-tagged recombinant Tt72 pol (82.7 kDa); Lane 1, lysate from *E. coli* Bl21(DE3)[pRARE, pET15_polTt72]; 2, the fraction of insoluble proteins; 3, the fraction of soluble proteins; 4, clear cell-lysate after centrifugation and heat-treatment (60 °C, 20 min); (5) HiTrap™ TALON affinity chromatography fraction; (6) HiTrap™ Heparin HP affinity chromatography fraction; (M) protein molecular mass markers (PageRuler™ Prestained Protein Ladder, Thermo Scientific).

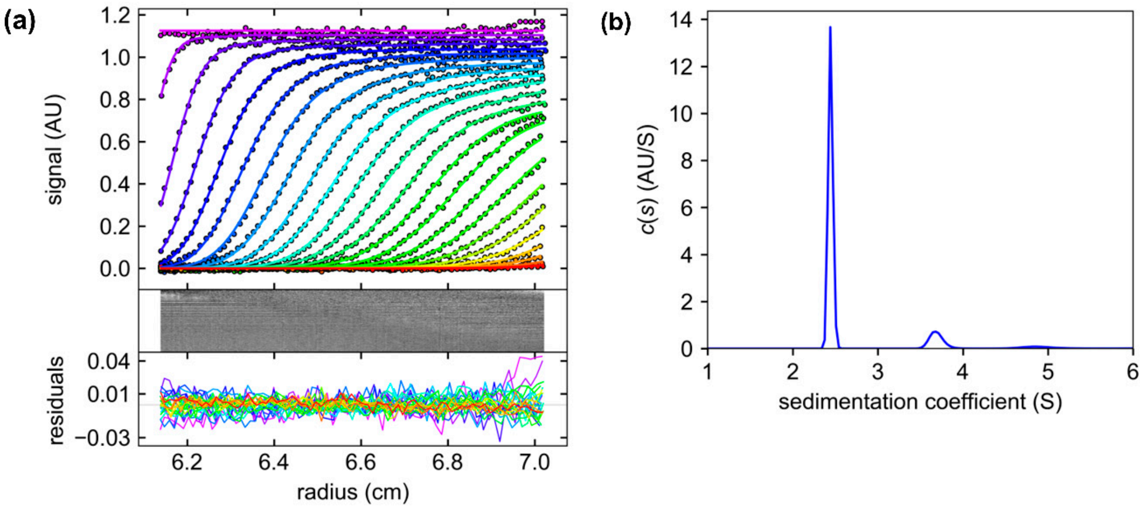


**Figure S3.** Determination of the Tt72 pol oligomeric state by analytical centrifugation. (**a**) Sedimentation-velocity data (dots) collected for Tt72 pol samples at concentration 1.0 mg/mL. Sedimentation velocity data were overlaid with the best-fit curves (lines) obtained from sedimentation coefficient distribution analysis. For clarity, only every third scan and every third data point is included. Below, the residuals of the experimental fits. (**b**) Sedimentation coefficient distributions [c(s)] of Tt72 pol.

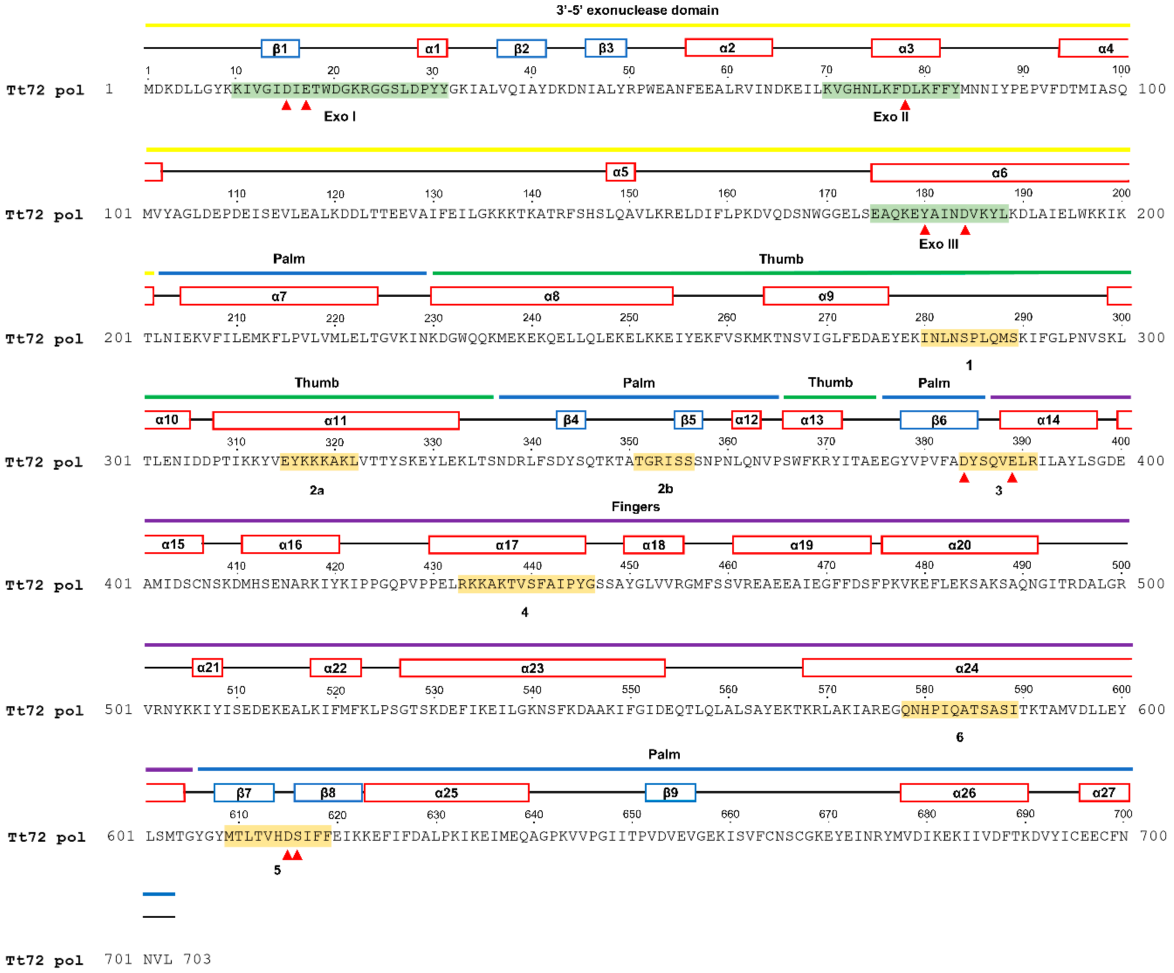


**Figure S4.** Secondary structure prediction of Tt72 pol. The amino acid sequence of Tt72 pol was subjected to secondary structure meta-server analysis [124], which was plotted as a line for coil, blue boxes for alpha-helices, and yellow boxes for beta-sheets. A colored line above the structural elements indicates the position of domains and subdomains. The yellow line indicates the 3′-5′ exo domain. Blue, green, and purple lines within the nucleotidyltransferase domain indicate palm, thumb, and fingers subdomains. The highly conserved motifs within the amino-acid sequence in both domains are indicated by colored unframed green and yellow boxes. The red triangle indicates the catalytic residues within the 3′-5′ exo and nucleotidyltransferase domain, which importance was tested using site-directed mutagenesis.

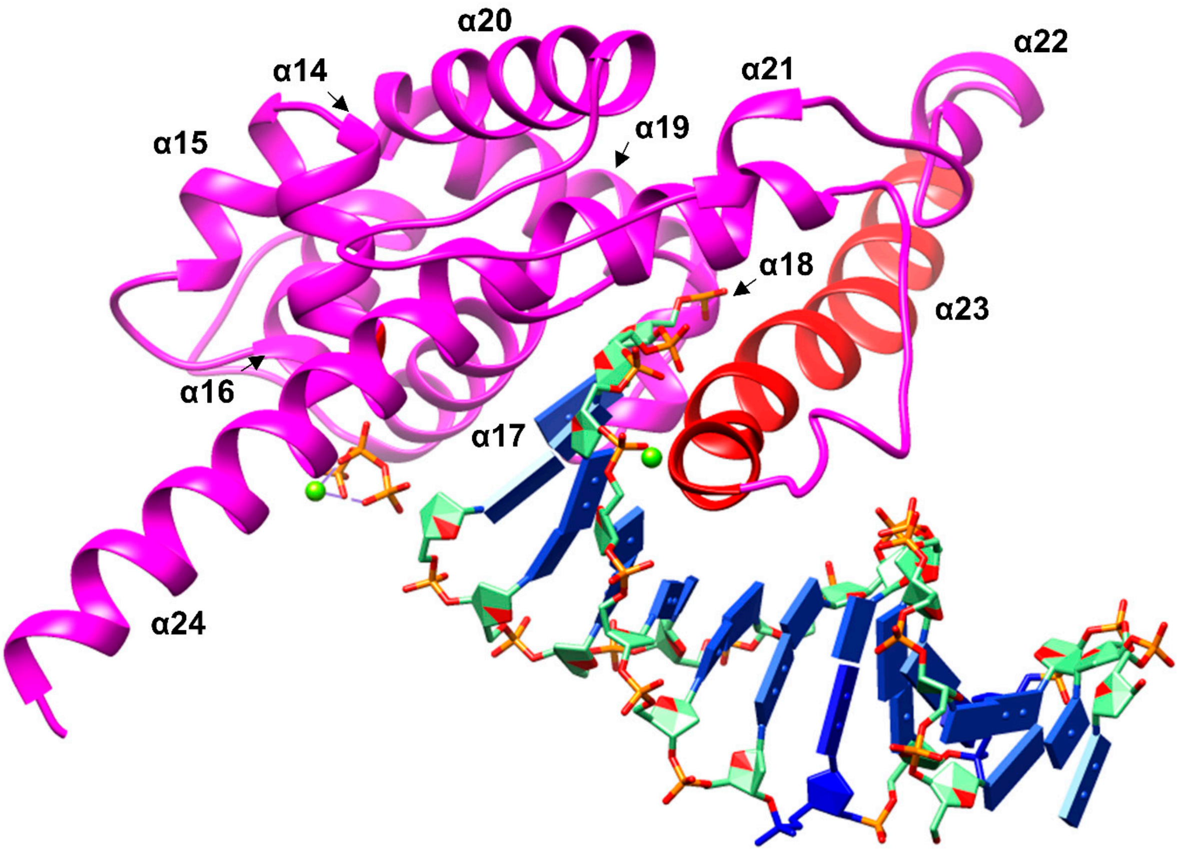


**Figure S5.** Structural model of subdomain fingers (387-605 aa) of Tt72 DNA polymerase. The unique secondary-structure element *α*-helix (*α*23, 527-553 aa) is shown in red.
